# Correlation between intestinal BMP2, IFNγ, and neural death in experimental infection with *Trypanosoma cruzi*

**DOI:** 10.1371/journal.pone.0246692

**Published:** 2021-02-09

**Authors:** José Rodrigues do Carmo Neto, Marcos Vinicius da Silva, Yarlla Loyane Lira Braga, Arthur Wilson Florencio da Costa, Simone Gonçalves Fonseca, Patricia Resende Alô Nagib, Mara Rúbia Nunes Celes, Milton Adriano Pelli Oliveira, Juliana Reis Machado

**Affiliations:** 1 Department of Bioscience and Technology, Institute of Tropical Pathology and Public Health, Federal University of Goias, Goiania, GO, Brazil; 2 Department of Microbiology, Immunology and Parasitology, Institute of Biological and Natural Sciences of Federal University of Triângulo Mineiro, Uberaba, Minas Gerais, Brazil; 3 Department of General Pathology, Federal University of Triângulo Mineiro, Uberaba, Minas Gerais, Brazil; Universidade Federal de Juiz de Fora, BRAZIL

## Abstract

Megacolon is one of the main late complications of Chagas disease, affecting approximately 10% of symptomatic patients. However, studies are needed to understand the mechanisms involved in the progression of this condition. During infection by *Trypanosoma cruzi* (*T*. *cruzi*), an inflammatory profile sets in that is involved in neural death, and this destruction is known to be essential for megacolon progression. One of the proteins related to the maintenance of intestinal neurons is the type 2 bone morphogenetic protein (BMP2). Intestinal BMP2 homeostasis is directly involved in the maintenance of organ function. Thus, the aim of this study was to correlate the production of intestinal BMP2 with immunopathological changes in C57Bl/6 mice infected with the *T*. *cruzi* Y strain in the acute and chronic phases. The mice were infected with 1000 blood trypomastigote forms. After euthanasia, the colon was collected, divided into two fragments, and a half was used for histological analysis and the other half for BMP2, IFNγ, TNF-α, and IL-10 quantification. The infection induced increased intestinal IFNγ and BMP2 production during the acute phase as well as an increase in the inflammatory infiltrate. In contrast, a decreased number of neurons in the myenteric plexus were observed during this phase. Collagen deposition increased gradually throughout the infection, as demonstrated in the chronic phase. Additionally, a BMP2 increase during the acute phase was positively correlated with intestinal IFNγ. In the same analyzed period, BMP2 and IFNγ showed negative correlations with the number of neurons in the myenteric plexus. As the first report of BMP2 alteration after infection by *T*. *cruzi*, we suggest that this imbalance is not only related to neuronal damage but may also represent a new route for maintaining the intestinal proinflammatory profile during the acute phase.

## Introduction

Bone morphogenetic proteins (BMPs), included in the transforming growth factor beta (TGF-β) superfamily, are involve from embryogenesis to adult organ cell homeostasis [1–5]. Initially described in bone formation [6], this protein group is divided into at least four subgroups with similar functions and biochemical structures [2]. One of the first reports of BMP2 was studied for its osteoinductive capacity. In addition, this protein is directly involved in intestinal homeostasis [7], intestine organogenesis, epithelial cell renovation, and myenteric plexus (MP) neuronal homeostasis [1, 7–9]. There is a network in the MP from which a muscular macrophage subpopulation (MMS) releases basal levels of BMP2 to neurons of this structure [7]. In response, these neurons produce the maintenance factor for the macrophage colony to regulate this MMS [7, 9]. This communication results in homeostasis of the gastrointestinal tract, and, when the MMS is depleted, BMP2 is not produced, leading to abnormal motility of the intestine [7].

BMP2 expression may be altered in diseases characterized by enteric nervous system (ENS) disturbances and altered intestinal motility. In Hirschsprung’s disease, BMP2 expression is upregulated compared to that in the normal gut [10]. In rats with induced diabetes, BMP2 expression is downregulated and returns to normal levels after insulin administration [11]. Despite differences in pathophysiology, both diseases are related to inflammation, neuronal loss, and intestinal dysmotility [7, 10–12]. It is reasonable that in infectious diseases that have neuronal damage, alterations in BMP2 levels could be associated with the progression of enteric nervous system pathophysiology, but there are no reports investigating this issue.

Three to ten percent of patients with chronic Chagas disease (CD) develop the digestive form, in which disturbances in the ENS lead to megacolon [13]. Currently, it is accepted that the progression of this form is related to neuronal loss due to infection, mainly due to the inflammatory process caused by *Trypanosoma cruzi* [14]. Observed differences in the inflammatory intestinal microenvironment between infected individuals without megacolon and those with megacolon suggest that inflammation effects the progression of the intestinal form. In the megacolon, the increases in eosinophils, mast cells, and macrophages are directly correlated with fibrosis in the organ, indicating they are participants in tissue damage [15]. Other cells, such as natural killer cells and cytotoxic T lymphocytes, have also been suggested as mediators of damage to ENS components [16]. In an experimental model, these same relationships have already been reported [17–19], including those regarding the participation of pro-inflammatory cytokines in the acute phase [20]. Thus, the inflammatory response generated by the host against *T*. *cruzi* needs to be extremely controlled, since in infected individuals without megacolon, there is less intestinal involvement and fewer tissue inflammatory cells [15, 16].

However, the molecular mechanisms underlying megacolon progression have not been elucidated. The intestinal myenteric plexus is the structure that is most affected by *T*. *cruzi* infection [21–23], with a broadly documented reduction of neuron cells [16, 23–26]. Since one of the most important functions of this structure is intestinal peristalsis regulation [27], neuronal damage leads to loss of intestinal homeostasis. It is known that neuropeptides, growth factors, and cytokines are directly involved in intestinal homeostasis, but so far little has been investigated in chagasic megacolon [28]. Since BMP2 can modulate and be modulated by the immune system and control intestinal homeostasis, the aim of this study was to correlate the production of intestinal BMP2 with cytokines and histopathological changes in mice infected with *T*. *cruzi* in different phases of experimental infection.

## Material and methods

### Animals

In the present study, male C57Bl/6 mice, aged 6 to 8 weeks, were used. Mice were either infected with the Y strain of *T*. *cruzi* or not infected, as a control. The animals were supplied by and maintained at the Bioterium of the Institute of Tropical Pathology and Public Health of the Federal University of Goias under controlled and known conditions: in plastic cages of 414 mm × 168 mm, temperature between 20 and 25°C, humidity between 45 and 55%, and with constant renewal of air and with a 12h photoperiod. They were fed with feed of known composition (Nuvilab-CR1, NUVITAL, Brazil) and were offered water *ad libitum*. All components of the cage, such as water, wood shavings, and feed, were autoclaved before use and exposure to the animals. The project was submitted to the Animal Use Ethics Committee of the Federal University of Goias and was approved under identification 051/19. The animals were monitored weekly until the date of euthanasia to minimize suffering as much as possible.

### Parasites and experimental infection

Blood trypomastigotes, maintained through serial passage in Balb/C mice, were inoculated in the mice destined for the experiment. Male C57Bl/6 mice were infected with 1000 trypomastigote forms of the Y strain of *T*. *cruzi*. After infection, mice were maintained for 30 days (acute phase) (n = 6) or 90 days (chronic phase) (n = 10). The chronic phase was characterized by blood parasitemia equal to zero and intestinal fibrosis. Non-infected control groups were maintained for the same amounts of time and under the same experimental conditions (30 days for acute phase with n = 5 and 90 days for chronic phase with n = 7). After acute or chronic phase development, mice were euthanized through cervical dislocation after confirmation of anesthetic status, induced by intraperitoneal administration of anesthetic solution at 5% xylazine hydrochloride and 10% ketamine. For all mice, two fragments of the colon were collected: the distal fragment for histological analysis and the proximal fragment for cytokine dosage. Throughout the study, there was no mortality. The experiment was repeated twice.

### Determination of parasitemia

The parasitemia of infected mice was carried out until no more circulating blood trypomastigotes were found. Through the caudal vein, 5 μL of blood was taken from the mice to assess the level of parasitemia. Under a slide and coverslip, parasites were counted in 50 random fields under an optical microscope at 400× magnification [29]. This observation was made every 3 days after the inoculum. The parasitemia result was normalized by the area of the slide and the area observed under the microscope according to the magnification used [30].

### Histopathological evaluations

The distal segment of the colon (comparable to the proximal two-thirds of the transverse colon in humans) of all groups was collected (approximately 1 cm), washed in cold saline solution, and fixed in 4% paraformaldehyde for 48 hours. Then, the material was dehydrated in an increasing series of ethyl alcohols, transferred to xylol, and then embedded in paraffin. The fragment was positioned on the longitudinal axis perpendicular to the microtomy plane. Serial 5 μm-thick sections were cut (e. g., every fifth section, with a gap of 25 μm between cuts) and selected sections were stained using H&E, Giemsa, and picrosirius red for intestinal analysis.

#### Inflammatory infiltrate quantification

Hematoxylin-eosin (HE)-stained slides were used to analyze the inflammatory infiltrate. From a light microscope attached to the camera, 10 micrographs (30 micrographs per animal) of each of the three serial fragments (100 μm apart) were captured under 400× magnification for further analysis. The intensity of the inflammatory process was assessed in the submucosal and muscular layers and classified as 0 (normal), 1 (discrete), 2 (moderate), and 3 (accentuated). After categorizing the photos, the mean of each case obtained was classified according to the following score: 0–0.3 = normal; 0.4–1.0 = discrete; 1.1–2 = moderate; 2.1–3 = accentuated (adapted from [31]).

#### Myenteric plexus neuron quantification

Giemsa-stained slides were used to quantify neurons. Four serial sections with 100 μm between each were evaluated under light microscopy at 400× magnification. This distance was selected so that a larger area could be analyzed. Neurons were counted in 30 random fields at 400× magnification in each cut (120 fields per animal). The result was expressed as the number of neurons/field.

#### Fibrous connective tissue quantification

Sirius-Red stained slides were used for morphometric evaluation of the deposition of connective tissue in the mucosa, submucosa, and intestinal muscle layers, except for the serous layer. Collagen analyses were carried out under polarized light and quantified using the Axion Vision software (ZEISS). For each intestinal fragment, 20 fields were analyzed at 400× magnification. The results were expressed as collagen (%) per unit area [32].

### Intestinal homogenates and cytokine measurements

The colon proximal fragment (approximately 1 cm) was transferred to an Eppendorf tube containing 1× Phosphate Buffered Saline solution and Complete ™ protease inhibitor (SIGMA, USA). Then, the fragments were subjected to homogenization in a homogenizer (DREMEL, EUA). The homogenates obtained were centrifuged at 12000 × *g* for 30 min, and the respective supernatants were stored at -80°C for quantification of BMP2, cytokines, and total proteins. The levels of BMP2, IFNγ, TNF-α, and IL-10 in the proximal intestine homogenates were measured by an enzyme-linked immunosorbent assay (ELISA). For BMP2, the commercial kit PEPROTECH (Lot # 0614T255) was used. For IFNγ (Lot # P209723) and TNF-α (Lot # P210424), the kit used was from the R&D System. Meanwhile, for IL-10, we used the BD OptEIA™ kit (Lot # 9164829). The methodologies were carried out according to the manufacturers’ instructions. For the colorimetric reaction, 3, 3, 5, 5-tetramethylbenzidine (TMB) (BD Pharmingen, USA) was used as a peroxidase substrate and the reading was made on a 450 nm filter in a microplate reader (Bio-Rad 2550 READER EIA, USA). The concentration of total proteins in the intestinal homogenate was determined using a NanoDrop spectrophotometer (Thermo Fisher Scientific, USA) and was used to normalize the concentrations of BMP2 and cytokines. The final concentration was given in pg/mg of tissue.

### Statistical analysis

Statistical analyses were performed using GraphPad Prism 6.0 (GraphPad Software—USA). The verification of the normal distribution of the quantitative variables was assessed by the Shapiro-Wilk test. For comparisons between two groups, an unpaired t-test was used for data with a normal distribution, and the Mann-Whitney test was used for data with a non-normal distribution. Correlations were made using the Spearman test with a 95% confidence interval. The results were considered statistically significant at p <0.05.

## Results

### Parasitemia

**Fig 1:**
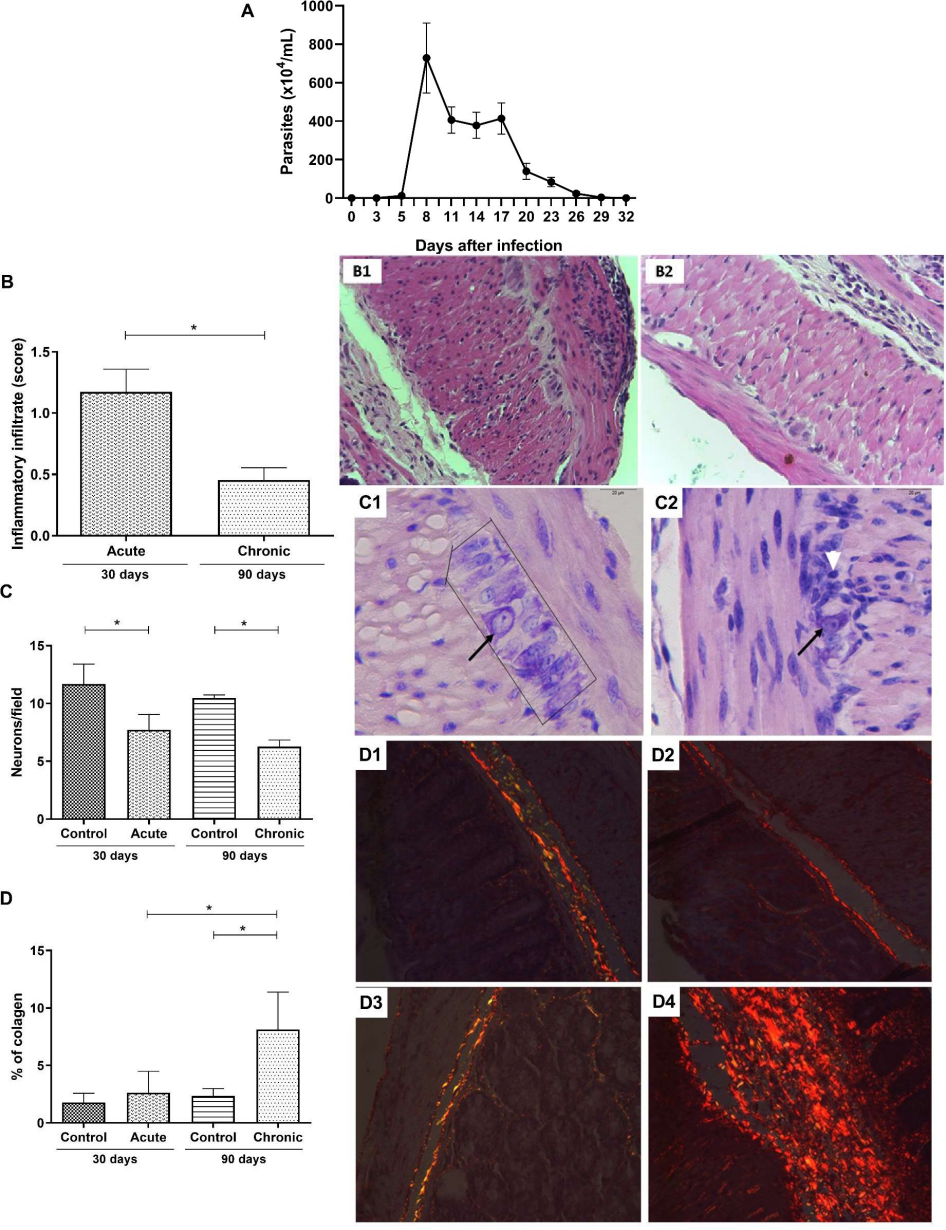
Blood parasitemia and intestinal histopathological differences between the acute and chronic phases of *T*. *cruzi* infected C57Bl/6 mice. C57Bl/6 mice were subcutaneously infected with 1000 blood trypomastigote forms of *T*. *cruzi* Y strain. (A) Parasitemia was determined by counting the number of trypomastigotes in 5 μl of blood collected from the caudal vein. (B) Intensity score of the intestinal inflammatory infiltrate. Intestinal photomicrographs of mice at the (B1) acute phase and (B2) chronic phase. (C) Number of neurons in the myenteric plexus. Intestinal photomicrographs of uninfected mice at 30 days (C1) and infected mice at the acute phase (C2). Neurons of the myenteric plexus are highlighted within black lines and highlighted by the black arrow. The white arrow represents an inflammatory infiltrate located close to the neurons of infected group mice during the acute phase. (D) Percentage of collagen deposited in the intestine. Intestinal photomicrographs of uninfected mice at (D1) 30 and (D2) 90 days and of infected mice during the (D3) acute and (D4) chronic phases. Mann-Whitney test. * Significant statistical differences at p < 0.05.

In order to monitor the infection by the *T*. *cruzi* Y strain in C57Bl/6 mice, parasitemia measurements were performed every three days after the inoculum. The analysis was carried out until the circulating blood trypomastigote forms were no longer found. Here, we observed a sustainable parasitemia in the acute phase, with detectable blood trypomastigote forms from the 5th to 32nd days (Fig 1A). Peak parasitemia was observed at 8 days of infection (Fig 1A). At the 29th day after infection, 93% of the animals showed reduced parasitemia. As expected, infected mice were able to control the blood parasite circulation at the end of the acute phase.

### Intestinal inflammation scores, neuronal quantification, and fibrosis deposition

To assess the extent of intestinal commitment in infected mice, the inflammatory infiltrate, neuronal loss, and collagen deposition were evaluated. The intestinal inflammatory infiltrate in the acute phase was more intense than that in the chronic phase (p = 0.0043) (Fig 1B). Most animals (approximately 80%) in the acute phase of infection showed a moderate inflammatory process (score: 1.1–1.5), whereas in the chronic phase, the inflammatory process decreased, showing a mild infiltrate for all animals (score: 0.3–0.6) (Fig 1B). The number of neurons was quantitated in the myenteric plexus to evaluate intestinal neural damage. A significant decrease in the number of neurons was detected in the acute phase of infection (30 days) (p = 0.0043), which was maintained during the chronic phase (90 days) (Fig 1C). No difference was found in the number of neurons between the acute and chronic phases (p = 0.0649). The same result was observed in the comparison between the controls (p = 0.2222). The last criterion for evaluating intestinal lesion progression was collagen deposition. As expected, no significant alteration in intestinal collagen deposition was observed in the acute phase; however, chronic infection induced a significant increase in collagen deposition when compared to the control group (p = 0.0001) and the acute phase group (p = 0.0017) (Fig 1D). No *T*. *cruzi* intestinal nests were found in either phase of the experimental infection in the HE intestinal sections.

### Experimental *T*. *cruzi* infection induces intestinal IFNγ and BMP2 upregulation in the acute phase

**Fig 2:**
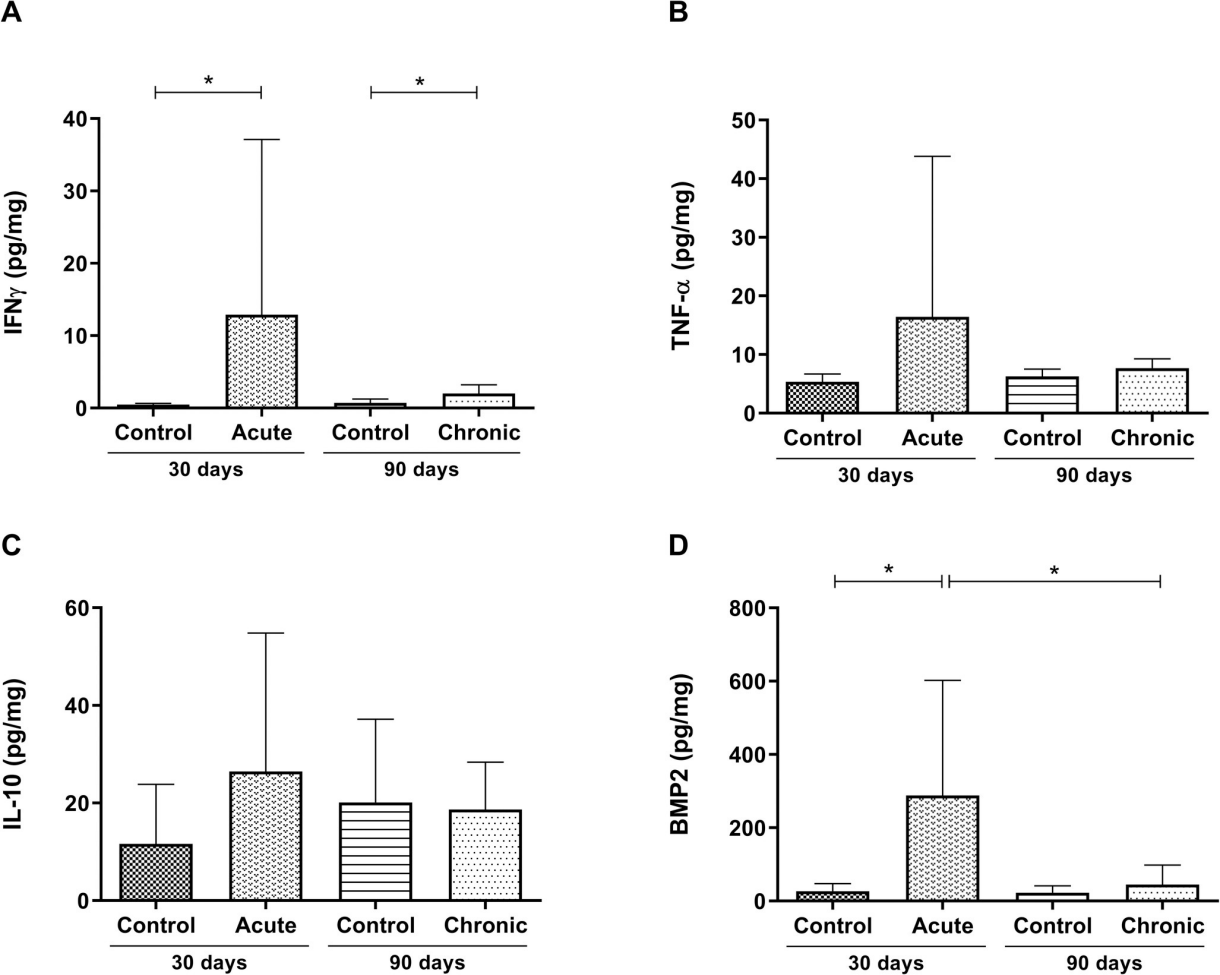
Intestinal immunological differences between the acute and chronic phases of *T*. *cruzi* infected C57Bl/6 mice. Quantification of the intestinal levels of (A) IFNγ, (B) TNF-α, (C) IL-10, and (D) BMP2 in picograms/mg. Data are presented for C57Bl/6 mice that were either not infected or infected with 1000 blood trypomastigote forms of the *T*. *cruzi* Y strain in the acute and chronic experimental phases of the disease. Mann-Whitney test. * Significant statistical differences at p <0.05.

In order to evaluate the immune response, the intestinal expression of cytokines was quantified at both experimental times. *T*. *cruzi* infection induced a significant increase in IFNγ production in both the acute phase (p = 0.0043) and the chronic phase (p = 0.0185) (Fig 2A). No statistical differences were found in the levels of intestinal TNF-α between infected and uninfected mice (Fig 2B). In addition, for both phases, no differences between the intestinal IL-10 levels of infected and uninfected mice were observed (Fig 2C), showing that infection was not regulated in this tissue. After confirming that the experimental model causes relevant histopathological and immunological changes, favoring intestinal inflammation, we evaluated whether BMP2 levels had also changed in the infected intestine. In the acute phase of infection, a significant increase in BMP2 production was observed compared to the control (p = 0.0303) (Fig 2D). In addition, the amount of BMP2 present in the intestine decreased in the chronic phase when compared to the acute phase (p = 0.0420), reaching the same level as that of the control basal production (Fig 2D).

### BMP2 is related to immunopathological changes only in the acute phase of experimental Chagas disease

**Fig 3:**
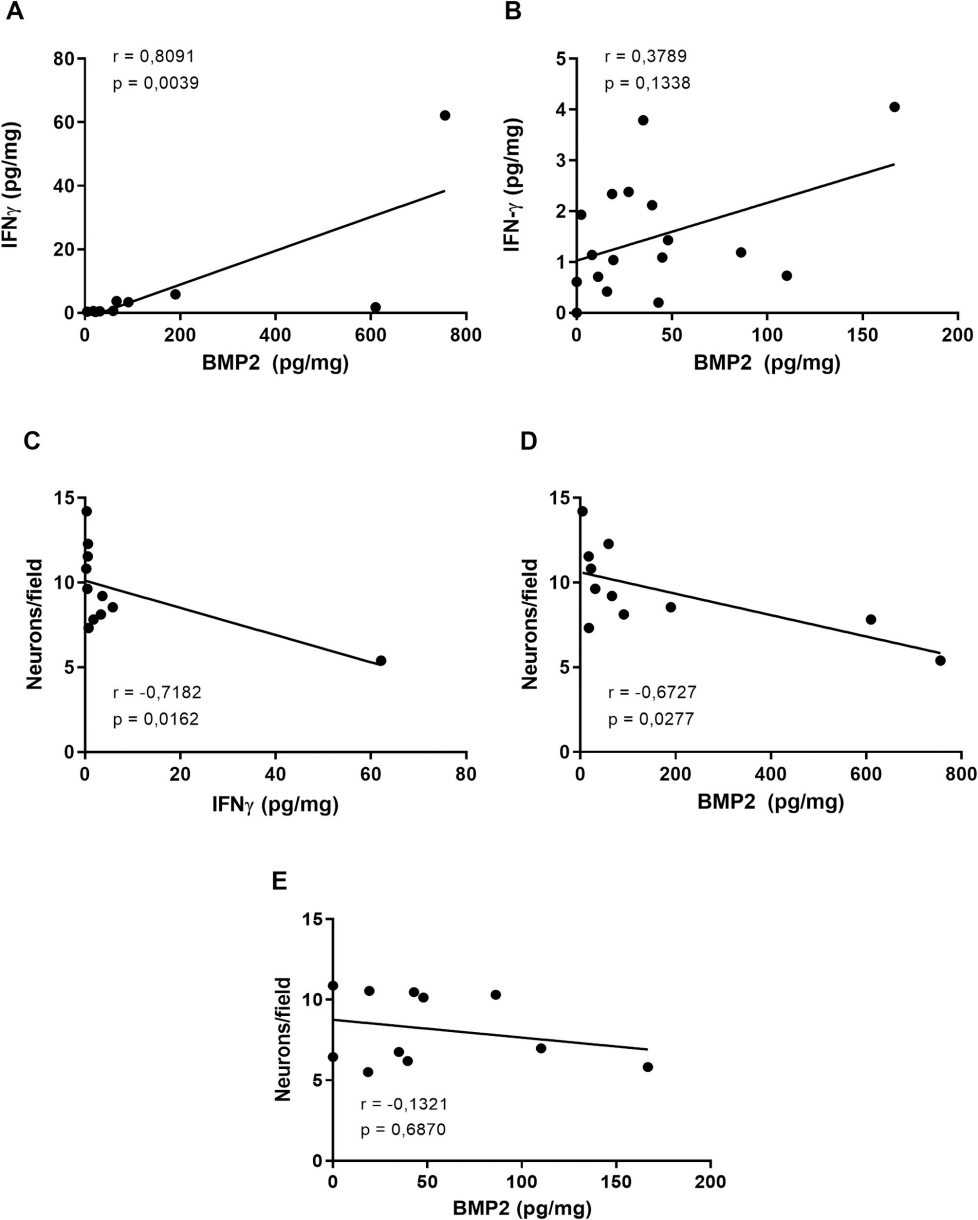
Correlations between intestinal IFNγ, BMP2, and myenteric neurons in the acute and chronic phases of *T*. *cruzi*-infected C57Bl/6 mice. Correlations between the intestinal IFNγ and BMP2 levels at the (A) acute and (B) chronic phases. Correlations between the number of neurons and the levels of intestinal (C) IFNγ in the acute phase, BMP2 in (D) the acute phase and (E) the chronic phase. The data presented are from C57Bl/6 mice infected with 1000 blood trypomastigote forms of *T*. *cruzi* Y strain. Correlations were performed using the Spearman test. Significant statistical differences at p < 0.05.

Since we observed, in the acute phase of the infection, significant BMP2, and IFNγ increases, we asked if IFNγ production could be correlated with the BMP2 increase. Interestingly, there was a strong, positive, and significant correlation between these two cytokines (Fig 3A) (p = 0.0039 and r = 0.8091, respectively). In the chronic phase, intestinal BMP2 returned to basal levels, abrogating the positive correlation (p = 0.1338 and r = 0.3789) (Fig 3B). For the next step, we asked whether these cytokines could be correlated with intestinal neuronal loss. There was a significant and strong and a moderate negative correlation between the number of neurons and the intestinal concentration of IFNγ (Fig 3C) and BMP2 (Fig 3D), respectively (p = 0.0162, r = -0.7182 and p = 0.0277, r = -0.6727) in the acute phase. No significant correlation was found between BMP2 and the number of intestinal neurons in the chronic phase (p = 0.6870, r = -0.1321) (Fig 3E).

## Discussion

This study aimed to investigate BMP2 intestinal production in C57Bl/6 mice infected with the Y strain of *T*. *cruzi* as well as to correlate this production with parameters related to the immunopathogenesis of the experimental infection. Our results demonstrate that infection induces the production of BMP2 differently in acute and chronic infections. A significant intestinal BMP2 increase was observed at 30 days of infection, and this was closely related to the intestinal IFNγ increment and neuronal loss. As this is the first report on intestinal BMP2 production in a protozoan disease, it is possible that there is a relationship between the imbalance we observed in the levels of this protein and the acute pathophysiology of intestinal CD.

It is already well established that strain Y parasitemia in experimental animals occurs quickly and in the first days after infection [20, 33], as observed in our study. The C57BL/6 mice is also well established in experiments related to an experimental CD, including in the study of intestinal changes [20, 34–37]. The intensity of the inflammatory infiltrate differs among infections caused by different strains and among different inocula of *T*. *cruzi* [20, 32, 38]. Independent of the strain used for murine infection, the acute phase is characterized by an intense inflammatory infiltrate when compared with the chronic phase [17, 31, 39], as was observed in our study. The inflammatory infiltrate was observed to be of greater intensity in the acute phase (moderate) than in the chronic phase (mild), which may be related to the fact that increased BMP2 levels were only observed at the beginning of the infection. In the stomachs of patients infected with *Helicobacter pylori*, it was observed that the inflammatory infiltrate induced by the infection produced BMP2, which was related to the increase of this protein in the organ [12]. In our study, the inflammatory infiltrate and BMP2 levels increased concomitantly. We believe that the inflammatory cells may influence the amount of BMP2 produced in the intestine during the acute phase. *In vitro*, BMP2 is able to induce chemotaxis, adhesion, and inhibit monocyte differentiation to the macrophage-M2 profile of human monocytes, which inhibits tissue repair and promotes the maintenance of the pro-inflammatory M1 profile, with an increase in pro-inflammatory molecules [40].

It was observed that BMP2 expression demonstrated a positive and significant correlation with IFNγ production in the intestine during the acute phase. Two mechanisms can be suggested from this result: 1) the increase in BMP2 was induced by the pro-inflammatory cytokines that increased in the acute phase and/or 2) BMP2 per se induced an increase in pro-inflammatory cytokines. Studies on bone regeneration have shown that BMP2 increases the production of pro-inflammatory cytokines in a dose-dependent manner [41–43]. In addition, stimulation of human endothelial cells (HMEC-1) with TNF-α resulted in an increase in the production of BMP2 [44]. The same authors also suggested a synergistic effect of these two cytokines (TNF-α and BMP2) in the establishment of the inflammatory process to induce an inflammatory phenotype in endothelial cells [44]. The ability of IFNγ and IL-1β to induce a BMP2 increase has also been demonstrated in the pancreatic cells of humans and murine models [45]. Although not evaluated in this study, the levels of pro-inflammatory cytokines, such as IL-12, IL-6, IL-2, and IL-17, have been reported to be elevated in the intestine during the acute experimental phase with the Y strain, in a manner that is dependent on the protozoan inoculum concentration [20]. In addition, it is suggested that the lack of balance between pro-inflammatory, regulatory, and anti-inflammatory cytokines results in the development and progression of CD [46–49]. The role of these cytokines is important both in the control of the protozoan [50–52] and in host tissue damage [34, 53]. Therefore, IFNγ and other pro-inflammatory cytokines could induce intestinal BMP2 upregulation, and vice versa. The interaction between IFNγ and BMP2 in maintaining the intestinal inflammatory profile during the acute phase of experimental *T*. *cruzi* infection, excludes the BMP2 participation in the chronic phase, in which there is a decrease in the inflammatory infiltrate, which results in decreased BMP2 production and no significant correlation with IFNγ in the intestine.

In experimental animals [54–56] and in humans [16, 21–23], *T*. *cruzi* infection decreases the number of nerve cells, and the acute phase has a leading role in this intensity [34, 57], as observed in our experimental model. The presence of inflammatory infiltrates together with pro-inflammatory cytokines, such as IFNγ, represents a way to contain the parasite during the initial events of *T*. *cruzi* infection [50]. In addition to attempting to control the infection, the production of IFNγ is related to neuronal decrease by inducing the production of nitric oxide via iNOS in an experimental model [34]. In our study, we found a strong negative correlation between the intestinal IFNγ levels and the number of neurons in the myenteric plexus, especially in the acute phase of experimental infection. While lymphocytes have already been demonstrated as a source of IFNγ [50, 51], natural killer cells and macrophages, which are at elevated levels in the chagasic megacolon [16], may also be responsible for the response to IFNγ (natural killer cells can produce IFNγ too) and produce NO, resulting in damage to the ENS. In fact, in an *in vitro* co-culture model with macrophages and neurons, phagocytes infected with *T*. *cruzi* and stimulated only with IFNγ induced neuronal death and neuritis by producing NO via iNOS [53]. In addition, the pathway associated with neuronal death in this model was attributed to necrosis of these cells [53]. Finally, when iNOS^-/-^ macrophages and NO blockers were used, neural death was mitigated by decreasing NO production, corroborating the neurotoxic action of this molecule [53]. Neurodegeneration has also been demonstrated *in vivo* in C57BL/6 mice IL-12/IL-23 (IL-12p40KO) deficient mice [58]. In this model, Bombeiro et al. (2010) suggested that IFNγ, together with the glia protein S100β, induces NO overproduction by both the inflammatory infiltrate and resident glial cells in the spinal cord [58]. In addition to necrosis, apoptosis and autophagy are also induced by NO in several neurological pathological conditions [58]. Moreover, IFNγ by itself is also considered neurotoxic because it induces neurons to produce NO via nNOS, an enzyme responsible for the production of neuronal NO [59] and neuronal toxicity [60]. Thus, these data corroborate the negative correlation between the IFNγ level and number of neurons found in our study, suggesting that one of the neuronal death pathways is dependent on IFNγ production.

In addition to the correlation involving IFNγ, a moderate, negative, and significant correlation was observed between the BMP2 level and the number of myenteric plexus neuron cells but only during the acute phase of the experimental infection. In fact, the imbalance in the production of this protein has already been observed to be related to changes in intestinal functionality and neuronal loss in a rat model of diabetes and in Hirschsprung’s disease [10, 11]. Furthermore, previous studies have demonstrated that an increase in BMP2 in neurons of the hippocampus of rats exposed to arsenic induced neuronal apoptosis via SMADs [61]. In addition, a high concentration of BMP2 *in vitro* has been shown to increase the proportion of apoptotic cells in primary cortical neurons [62]. Thus, we speculate that the BMP2 increase represents a new pathway for neuronal death, along with IFNγ, in the intestinal form of experimental CD during the acute phase, a determining moment for the decrease of nerve cells.

Although there is a reduction in the inflammatory process in the chronic phase, tissue damage continues to progress, as observed in our model, through the increasing deposition of intestinal collagen after 90 days of infection. The mechanisms involved in the progression of CD are still debated. The parasitic load at the beginning of the infection, integration of kDNA into the host cells, (auto)immune response, and the persistence of the protozoans are factors that are collectively related to tissue damage, which over long time periods results in cell destruction, followed by fibrosis, and ultimately loss of tissue function in the most severe forms of CD [31, 63–66]. Although it is accepted that *T*. *cruzi* infection induces an inflammatory process associated with neuronal death, other components, such as the destruction of Cajal cells, smooth muscle cells [67], and enteric glial cells [16], are also related to the progression of megacolon and loss of motility and intestinal function.

BMP2 can also represent another component related to the intestinal fibrotic process that has not yet been evaluated in CD. In experimental models of chronic pancreatitis [68], renal fibrosis [69], and pulmonary fibrosis [70], BMP2 has been reported to have anti-fibrotic properties, mainly related to inhibition of TGF-β-related pathways. Based on this information, we speculate that, at the beginning of the infection, the increase in BMP2 is related to the inhibition of intestinal remodeling in our model. In the chronic phase, in which there is no BMP2, collagen deposition occurs.

From the results obtained in this study, some hypotheses can be developed regarding the role of BMP2 in experimental CD. This protein can act in several ways to participate in the progression of intestinal changes observed during *T*. *cruzi* infection. Briefly, we propose that, during the acute phase of infection, the increase in BMP2 is related to 1) the increase in inflammatory cells producing BMP2, 2) the maintenance of the intestinal proinflammatory profile, and 3) neuronal destruction. However, it is also plausible that, by inhibiting intestinal remodeling in the early stages of experimental infection, BMP2 plays a dual role. These findings may represent a new mechanism of interaction between the immune and nervous systems in the progression of Chagas disease.

## Supporting information

S1 File (XLSX)

## References

[1] Goldstein AM, Brewer KC, Doyle AM, Nagy N, Roberts DJ. BMP signaling is necessary for neural crest cell migration and ganglion formation in the enteric nervous system. Mech Dev. 2005;122(6):821–33. 10.1016/j.mod.2005.03.003 15905074

[2] Bragdon B, Moseychuk O, Saldanha S, King D, Julian J, Nohe A. Bone Morphogenetic Proteins: A critical review. Cell Signal. 2011 4;23(4):609–20. 10.1016/j.cellsig.2010.10.003 20959140

[3] Chalazonitis A, D’Autréaux F, Pham TD, Kessler JA, Gershon MD. Bone morphogenetic proteins regulate enteric gliogenesis by modulating ErbB3 signaling. Dev Biol. 2011 2;350(1):64–79. 10.1016/j.ydbio.2010.11.017 21094638 PMC3034360

[4] Kashima R, Hata A. The role of TGF-β superfamily signaling in neurological disorders. Acta Biochim Biophys Sin (Shanghai). 2018;50(1):106–20. 10.1093/abbs/gmx124 29190314 PMC5846707

[5] Ampuja M, Kallioniemi A. Transcription factors—Intricate players of the bone morphogenetic protein signaling pathway. Genes Chromosom Cancer. 2018;57(1):3–11. 10.1002/gcc.22502 28857319

[6] Chen D, Zhao M, Mundy GR. Bone Morphogenetic Proteins. Growth Factors. 2004 12;22(4):233–41. 10.1080/08977190412331279890 15621726

[7] Muller PA, Koscsó B, Rajani GM, Stevanovic K, Berres ML, Hashimoto D, et al Crosstalk between muscularis macrophages and enteric neurons regulates gastrointestinal motility. Cell. 2014;158(2):300–13. 10.1016/j.cell.2014.04.050 25036630 PMC4149228

[8] Chen K, Xie W, Luo B, Xiao W, Teitelbaum DH, Yang H, et al Intestinal mucosal barrier is injured by BMP2/4 via activation of NF- B signals after ischemic reperfusion. Mediators Inflamm. 2014;2014 10.1155/2014/901530 25132736 PMC4124715

[9] Locati M. Macrophages Have a Grip on the Gut. Immunity. 2014;41(1):11–3. 10.1016/j.immuni.2014.07.002 25035949

[10] Wu M, Chen W, Mi J, Chen D, Wang W, Gao H. Expression analysis of BMP2, BMP5, BMP10 in human colon tissues from Hirschsprung disease patients. Int J Clin Exp Pathol. 2014;7(2):529–36. 24551273 PMC3925897

[11] Honoré SM, Zelarayan LC, Genta SB, Sánchez SS. Neuronal loss and abnormal BMP/Smad signaling in the myenteric plexus of diabetic rats. Auton Neurosci Basic Clin. 2011;164(1–2):51–61. 10.1016/j.autneu.2011.06.003 21737358

[12] Bleuming SA, Kodach LL, Garcia Leon MJ, Richel DJ, Peppelenbosch MP, Reitsma PH, et al Altered bone morphogenetic protein signalling in the Helicobacter pylori-infected stomach. J Pathol. 2006;209(2):190–7. 10.1002/path.1976 16550632

[13] Adad SJ, Barbosa E Silva G, Jammal AA. The significantly reduced number of interstitial cells of Cajal in chagasic megacolon (CM) patients might contribute to the pathophysiology of CM. Virchows Arch. 2012;461(4):385–92. 10.1007/s00428-012-1299-7 22895866

[14] Oliveira EC1, Freitas MAR2, Brehmer A3 DSA. Immunological Challenges to the Development of Chagasic Mega Syndromes. Int J Cell Sci Mol Biol [Internet]. 2018;3(5).

[15] da Silveira ABM, Adad SJ, Correa-Oliveira R, Furness JB, D’Avila Reis D. Morphometric study of eosinophils, mast cells, macrophages and fibrosis in the colon of chronic chagasic patients with and without megacolon. Parasitology. 2007;134(6):789–96. 10.1017/S0031182007002296 17288632

[16] da Silveira ABM, Lemos EM, Adad SJ, Correa-Oliveira R, Furness JB, D’Avila Reis D. Megacolon in Chagas disease: a study of inflammatory cells, enteric nerves, and glial cells. Hum Pathol [Internet]. 2007;38(8):1256–64. 10.1016/j.humpath.2007.01.020 17490721

[17] Campos CF, Cangussú SD, Duz ALC, Cartelle CT, De Lourdes Noviello M, Veloso VM, et al Enteric neuronal damage, intramuscular denervation and smooth muscle phenotype changes as mechanisms of Chagasic megacolon: Evidence from a long-term murine model of tripanosoma cruzi infection. PLoS One. 2016;11(4):1–18. 10.1371/journal.pone.0153038 PMC4821538 27045678

[18] Molina HA, Kierszenbaum F. Interaction of human eosinophils or neutrophils with *Trypanosoma cruzi* in vitro causes bystander cardiac cell damage. Immunology. 1989;66(2):289–95. 2647628 PMC1385102

[19] Kannen V, Sakita JY, Carneiro ZA, Bader M, Alenina N, Teixeira RR, et al Mast Cells and Serotonin Synthesis Modulate Chagas Disease in the Colon: Clinical and Experimental Evidence. Dig Dis Sci. 2018;63(6):1473–84. 10.1007/s10620-018-5015-6 29569002

[20] Vazquez BP, Vazquez TP, Miguel CB, Rodrigues WF, Mendes MT, De Oliveira CJF, et al Inflammatory responses and intestinal injury development during acute *Trypanosoma cruzi* infection are associated with the parasite load. Parasites and Vectors. 2015;8(1):206 10.1186/s13071-015-0811-8 25889515 PMC4399205

[21] Tafuri WL. Light and electron microscope studies of the autonomic nervous system in experimental and human American trypanosomiasis. Virchows Arch Abteilung A Pathol Anat. 1971;354(2):136–49. 10.1007/BF00548079 5000755

[22] Adad SJ, Andrade DC, Lopes ER, Chapadeiro E. Pathological anatomy of chagasic megaesophagus. Rev Inst Med Trop Sao Paulo. 1991;33(6):443–50. 1844974

[23] Adad SJ, Cançado CG, Etchebehere RM, Teixeira VPA, Gomes UA, Chapadeiro E, et al Neuron count reevaluation in the myenteric plexus of chagasic megacolon after morphometric neuron analysis. Virchows Arch. 2001;438(3):254–8. 10.1007/s004280000319 11315622

[24] da SILVEIRA ABM, ARANTES RME, VAGO AR, LEMOS EM, ADAD SJ, CORREA-OLIVEIRA R, et al Comparative study of the presence of *Trypanosoma cruzi* kDNA, inflammation and denervation in chagasic patients with and without megaesophagus. Parasitology. 2005;131(5):627–34. 10.1017/S0031182005008061 16255821

[25] Maifrino LBM, Amaral SON, Watanabe I, Liberti EA, Souza RR De. *Trypanosoma cruzi*: Preliminary investigation of NADH-positive and somatostatin–immunoreactive neurons in the myenteric plexus of the mouse colon during the infection. 2005;111:224–9. 10.1016/j.exppara.2005.08.008 16202412

[26] Kramer K, Alexandre BM, Brehmer A. Quantitative evaluation of neurons in the mucosal plexus of adult human intestines. 2011;1–9. 10.1007/s00418-011-0807-1 21461752

[27] Furness JB. The enteric nervous system and neurogastroenterology. Nat Rev Gastroenterol Hepatol; 2012;9: 286–94. 10.1038/nrgastro.2012.32 22392290

[28] Freitas MAR, Segatto N, Pedro, Remolli F, Oliveira EC de, Jabari S, et al Neurotrophin Expression in Chagasic Megacolon. 2017;1(3):1013

[29] BRENER Z. Therapeutic activity and criterion of cure on mice experimentally infected with *Trypanosoma cruzi*. Rev Inst Med Trop Sao Paulo. 1962;4:389–96. 14015230

[30] Araújo-Jorge TC de, Castro SL de. Doença de chagas: manual para experimentação animal. Doença de chagas: manual para experimentação animal. Editora FIOCRUZ; 2000.

[31] Wesley M, Moraes A, Rosa A de C, Carvalho JL, Shiroma T, Vital T, et al Correlation of parasite burden, kdna integration, autoreactive antibodies, and cytokine pattern in the pathophysiology of chagas disease. Front Microbiol. 2019;10(AUG):1856. 31496999 10.3389/fmicb.2019.01856 PMC6712995

[32] Reis Machado J, Silva MV, Borges DC, Da Silva CA, Ramirez LE, Dos Reis MA, et al Immunopathological aspects of experimental *Trypanosoma cruzi* reinfections. Biomed Res Int. 2014;2014: 648715 10.1155/2014/648715 25050370 PMC4094717

[33] Souza NDD, Belin BS, Massocatto CL, Araújo SMD, Sant’ana DMG, Araújo EJA, et al Effect of acetylsalicylic acid on total myenteric neurons in mice experimentally infected with *Trypanosoma cruzi*. An Acad Bras Cienc. 2019;91(2):e20180389 10.1590/0001-3765201920180389 31141012

[34] Arantes RME, Marche HHF, Bahia MT, Cunha FQ, Rossi MA, Silva JS. Interferon-γ-Induced Nitric Oxide Causes Intrinsic Intestinal Denervation in *Trypanosoma cruzi*-Infected Mice. Am J Pathol. 2004;164(4):1361–8. 10.1016/s0002-9440(10)63222-1 15039223 PMC1615344

[35] De Souza AP, Sieberg R, Li H, Cahill HR, Zhao D, Araújo-Jorge TC, et al The role of selenium in intestinal motility and morphology in a murine model of Typanosoma cruzi infection. Parasitol Res. 2010;106(6):1293–8. 10.1007/s00436-010-1794-1 20195635 PMC2861723

[36] Andrade LO, Machado CRS, Chiari E, Pena SDJ, Macedo AM. *Trypanosoma cruzi*: Role of host genetic background in the differential tissue distribution of parasite clonal populations. Exp Parasitol. 2002;100(4):269–75. 10.1016/s0014-4894(02)00024-3 12128054

[37] Pereira N de S, Queiroga TBD, da Silva DD, Nascimento MSL, Andrade CM de, Souto JT de, et al NOD2 receptor is crucial for protecting against the digestive form of Chagas disease. PLoS Negl Trop Dis. 2020;14(9):e0008667 10.1371/journal.pntd.0008667 32986710 PMC7553797

[38] Espinoza B, Solorzano-Domínguez N, Vizcaino-Castillo A, Martínez I, Elias-López AL, Rodríguez-Martínez JA. Gastrointestinal infection with Mexican TcI *Trypanosoma cruzi* strains: Different degrees of colonization and diverse immune responses. Int J Biol Sci. 2011;7(9):1357–70. 10.7150/ijbs.7.1357 22110387 PMC3221943

[39] Mateus J, Guerrero P, Lasso P, Cuervo C, González JM, Puerta CJ, et al An animal model of acute and chronic chagas disease with the reticulotropic Y strain of *Trypanosoma cruzi* that depicts the multifunctionality and dysfunctionality of T cells. Front Immunol. 2019;10(APR):918. 31105709 10.3389/fimmu.2019.00918 PMC6499084

[40] Pardali E, Makowski LM, Leffers M, Borgscheiper A, Waltenberger J. BMP-2 induces human mononuclear cell chemotaxis and adhesion and modulates monocyte-to-macrophage differentiation. J Cell Mol Med. 2018;22(11):5429–38. 10.1111/jcmm.13814 30102472 PMC6201342

[41] Zara JN, Siu RK, Zhang X, Shen J, Ngo R, Lee M, et al High doses of bone morphogenetic protein 2 induce structurally abnormal bone and inflammation in vivo. Tissue Eng—Part A. 2011;17(9–10):1389–99. 10.1089/ten.TEA.2010.0555 21247344 PMC3079169

[42] Lee KB, Taghavi CE, Song KJ, Sintuu C, Yoo JH, Keorochana G, et al Inflammatory characteristics of rhBMP-2 in vitro and in an in vivo rodent model. Spine (Phila Pa 1976). 2011;36(3):E149–54. 21242879 10.1097/BRS.0b013e3181f2d1ec

[43] Mitchell K, Shah JP, Dalgard CL, Tsytsikova L V., Tipton AC, Dmitriev AE, et al Bone morphogenetic protein-2-mediated pain and inflammation in a rat model of posterolateral arthrodesis. BMC Neurosci. 2016;17(1):80 10.1186/s12868-016-0314-3 27905881 PMC5134101

[44] Sanders LN, Schoenhard JA, Saleh MA, Mukherjee A, Ryzhov S, McMaster WG, et al BMP antagonist gremlin 2 limits inflammation after myocardial infarction. Circ Res. 2016;119(3):434–49. 10.1161/CIRCRESAHA.116.308700 27283840 PMC4961528

[45] Ibarra Urizar A, Friberg J, Christensen DP, Lund Christensen G, Billestrup N. Inflammatory Cytokines Stimulate Bone Morphogenetic Protein-2 Expression and Release from Pancreatic Beta Cells. J Interf Cytokine Res. 2016;36(1):20–9. 10.1089/jir.2014.0199 26308798

[46] Da Silva MV, De Almeida VL, De Oliveira WD, Matos Cascudo NC, De Oliveira PG, Da Silva CA, et al Upregulation of Cardiac IL-10 and Downregulation of IFN-γ in Balb/c IL-4 −/− in Acute Chagasic Myocarditis due to Colombian Strain of *Trypanosoma cruzi*. Mediators Inflamm. 2018;2018:3421897 10.1155/2018/3421897 30622430 PMC6304210

[47] D’Ávila DA, Guedes PMM, Castro AM, Gontijo ED, Chiari E, Galvão LMC. Immunological imbalance between IFN-γ and IL-10 levels in the sera of patients with the cardiac form of Chagas disease. Mem Inst Oswaldo Cruz. 2009;104(1):100–5. 10.1590/s0074-02762009000100015 19274383

[48] Cunha-Neto E, Chevillard C. Chagas disease cardiomyopathy: Immunopathology and genetics. Mediators Inflamm. 2014;2014:683230 10.1155/2014/683230 25210230 PMC4152981

[49] Guedes PMM, Gutierrez FRS, Silva GK, Dellalibera-Joviliano R, Rodrigues GJ, Bendhack LM, et al Deficient regulatory T cell activity and low frequency of IL-17-producing T cells correlate with the extent of cardiomyopathy in human Chagas’ disease. PLoS Negl Trop Dis. 2012;6(4):e1630 10.1371/journal.pntd.0001630 22545173 PMC3335880

[50] Gazzinelli RT, Oswald IP, Hieny S, James SL, Sher A. The microbicidal activity of interferon‐γ‐treated macrophages against *Trypanosoma cruzi* involves an L‐arginine‐dependent, nitrogen oxide‐mediated mechanism inhibitable by interleukin‐10 and transforming growth factor‐β. Eur J Immunol. 1992;22(10):2501–6. 10.1002/eji.1830221006 1396957

[51] Vespa GNR, Cunha FQ, Silva JS. Nitric oxide is involved in control of *Trypanosoma cruzi*-induced parasitemia and directly kills the parasite in vitro. Infect Immun. 1994;62(11):5177–82. 10.1128/IAI.62.11.5177-5182.1994 7523307 PMC303244

[52] Miyazaki Y, Hamano S, Wang S, Shimanoe Y, Iwakura Y, Yoshida H. IL-17 Is Necessary for Host Protection against Acute-Phase *Trypanosoma cruzi* Infection. J Immunol. 2010;185(2):1150–7. 10.4049/jimmunol.0900047 20562260

[53] Megale de Almeida-Leite C, da Cunha Galvão LM, Afonso LCC, de Queiróz Cunha F, Arantes RME. Interferon-γ induced nitric oxide mediates in vitro neuronal damage by *Trypanosoma cruzi*-infected macrophages. Neurobiol Dis. 2007;25(1):170–8. 10.1016/j.nbd.2006.09.003 17056264

[54] Machado EMM, Camilo Júnior DJ, Pinheiro SW, Lopes ER, Fernandes AJ, Dias JCP, et al Morphometry of Submucous and Myenteric Esophagic Plexus of Dogs Experimentally Reinfected with *Trypanosoma cruzi*. Mem Inst Oswaldo Cruz. 2001;96(4):545–8. 10.1590/s0074-02762001000400017 11391429

[55] Moreira NM, Sant’ana DMG, Araújo EJA, Toledo MJO, Gomes ML, de Araújo SM. Neuronal changes caused by *Trypanosoma cruzi*: An experimental model. An Acad Bras Cienc. 2011;83(2):545–55. 10.1590/s0001-37652011000200014 21670878

[56] Moreira NM, Santos FN, Jean M, Toledo O, Moraes SMF, Araujo EJA, et al Moderate physical exercise reduces parasitaemia and protects colonic myenteric neurons in mice infected with *Trypanosoma cruzi*. Int J Exp Pathol. 2013;94(6):426–35. 10.1111/iep.12049 24205797 PMC3944454

[57] De Souza MM, Andrade SG, Barbosa AA, Santos RTM, Alves VAF, Andrade ZA. *Trypanosoma cruzi* Strains and Autonomic Nervous System Pathology in Experimental Chagas Disease. Mem Inst Oswaldo Cruz. 1996;91(2):217–24. 10.1590/s0074-02761996000200018 8736094

[58] Bombeiro AL D’Império Lima MR, Chadi G, Álvarez JM. Neurodegeneration and increased production of nitrotyrosine, nitric oxide synthase, IFN-γ and s100β protein in the spinal cord of IL-12p40-deficient mice infected with *Trypanosoma cruzi*. Neuroimmunomodulation. 2010;17(2):67–78. 10.1159/000258689 19923851

[59] Mizuno T, Zhang G, Takeuchi H, Kawanokuchi J, Wang J, Sonobe Y, et al Interferon‐γ directly induces neurotoxicity through a neuron specific, calcium‐permeable complex of IFN‐γ receptor and AMPA GluRl receptor. FASEB J. 2008;22(6):1797–806. 10.1096/fj.07-099499 18198214

[60] Dawson TM, Dawson VL. Nitric Oxide Signaling in Neurodegeneration and Cell Death. Adv Pharmacol. 2018;82:57–83. 10.1016/bs.apha.2017.09.003 29413528

[61] Pandey R, Rai V, Mishra J, Mandrah K, Kumar Roy S, Bandyopadhyay S. From the Cover: Arsenic Induces Hippocampal Neuronal Apoptosis and Cognitive Impairments via an Up-Regulated BMP2/Smad-Dependent Reduced BDNF/TrkB Signaling in Rats. Toxicol Sci. 2017;159(1):137–58. 10.1093/toxsci/kfx124 28903487

[62] Sun Q, Mao S, Li H, Zen K, Zhang CY, Li L. Role of miR-17 family in the negative feedback loop of bone morphogenetic protein signaling in neuron. PLoS One. 2013;8(12):e83067 10.1371/journal.pone.0083067 24349434 PMC3859655

[63] Scharfstein J, Gomes J de AS, Correa-Oliveira R. Back to the future in Chagas disease: From animal models to patient cohort studies, progress in immunopathogenesis research. Mem Inst Oswaldo Cruz. 2009;104(SUPPL. 1):187–98. 10.1590/s0074-02762009000900025 19753474

[64] Jabari S, De Oliveira EC, Brehmer A, Da Silveira ABM. Chagasic megacolon: Enteric neurons and related structures. Histochem Cell Biol. 2014;142(3):235–44. 10.1007/s00418-014-1250-x 25059649 PMC4133073

[65] De Bona E, Lidani KCF, Bavia L, Omidian Z, Gremski LH, Sandri TL, et al Autoimmunity in chronic chagas disease: A road of multiple pathways to cardiomyopathy?. Front Immunol. 2018;9:1842 10.3389/fimmu.2018.01842 30127792 PMC6088212

[66] Bonney KM, Luthringer DJ, Kim SA, Garg NJ, Engman DM. Pathology and Pathogenesis of Chagas Heart Disease. Annu Rev Pathol. 2019;14:421–447 10.1146/annurev-pathol-020117-043711 30355152 PMC7373119

[67] Jabari S, da Silveira ABM, de Oliveira EC, Quint K, Wirries A, Neuhuber W, et al Interstitial cells of Cajal: Crucial for the development of megacolon in human Chagas’ disease? Color Dis. 2013;15(10):e592–8. 10.1111/codi.12331 23810202

[68] Gao X, Cao Y, Yang W, Duan C, Aronson JF, Rastellini C, et al BMP2 inhibits TGF-β-induced pancreatic stellate cell activation and extracellular matrix formation. Am J Physiol Gastrointest Liver Physiol. 2013;304(9):G804–13. 10.1152/ajpgi.00306.2012 23429583 PMC3652003

[69] Yang YL, Liu YS, Chuang LY, Guh JY, Lee TC, Liao TN, et al Bone morphogenetic protein-2 antagonizes renal interstitial fibrosis by promoting catabolism of type i transforming growth factor-β receptors. Endocrinology. 2009;150(2):727–40. 10.1210/en.2008-0090 18832104

[70] Myllärniemi M, Lindholm P, Ryynänen MJ, Kliment CR, Salmenkivi K, Keski-Oja J, et al Gremlin-mediated decrease in bone morphogenetic protein signaling promotes pulmonary fibrosis. Am J Respir Crit Care Med. 2008;177(3):321–9. 10.1164/rccm.200706-945OC 17975199 PMC2218851

